# St. Thomas's Hospital

**Published:** 1899-05-13

**Authors:** 


### ST. THOMAS'S HOSPITAL.

### OPENING OF THE NEW ACCIDENT WARD.

The Lord Mayor of London, accompanied by the Lady Mayoress, paid a state visit to St. Thomas's Hospital on the 4th inst. to open the new accident ward. It is appropriately named the City of London Ward, and it has been established mainly by the munificence of the Mercers' Company, who in 1897 presented £10,000 in commemoration of the Diamond Jubilee. The Treasurer (Mr. J. G. Wainwright), in addressing the Lord Mayor, stated that the records of the hospital proved that as far back as the reign of Stephen it was endeavouring to carry comfort and help to the sick and the unfortunate. It was originally located in Southwark, but the ancient site had to be absorbed for railway purposes, and it became necessary to find a new position. At the time that the present site was decided upon it was most adversely criticised in some quarters, but the wisdom of the choice has since been shown, for it has proved to be admirably adapted for the work of the institution. Unfortunately, the hospital revenues by reason of the depression in agriculture had been seriously affected, and for some twenty years one-fourth of the beds had to remain unoccupied. But the result of a Mansion House Fund in 1895, together with a grant of £1,800 a year from the Prince of Wales's Fund, had enabled the governors to open three of the closed wards. "It was much hoped," continued the Treasurer, "that the noble example of the Mercers' Company might be followed, so that the ward about to be opened might in time be in reality a City of London ward, not merely carrying its name, but, by the liberality of the citizens, endowed by it with the necessary income." The Lord Mayor, in replying, also spoke in terms of admiration of the present site which, he said, was situated close to the teeming population which the hospital was built to serve, was in a position to enjoy the advantage of plenty of light and air, and also to appeal to the sympathies of those who passed by.

After prayer had been offered by the Bishop of Rochester, a procession was formed, and the civic party, the bishops and clergy, the hospital staff, and the visitors inspected the new ward which had been fitted with every modern requirement, and should prove a great boon to the patients, as well as a great convenience to the staff.

